# Supplementary material for: Evaluation of Existing Models to Estimate Sorption Coefficients for Ionisable Pharmaceuticals in Soils and Sludge
Source: Toxics. 2020 Feb 11;8(1):13. doi: 10.3390/toxics8010013 (PMC7151744; doi:10.3390/toxics8010013)
Supplement: Supplementary file 1 [file toxics-08-00013-s001.pdf]

Supplementary Materials: Evaluation of Existing Models to Estimate Sorption Coefficients for Ionisable Pharmaceuticals in Soils and Sludge

Laura J. Carter, John L. Wilkinson and Alistair B.A. Boxall

(a)

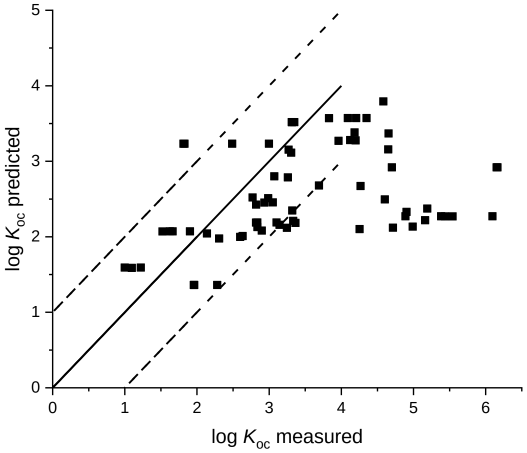

(b)

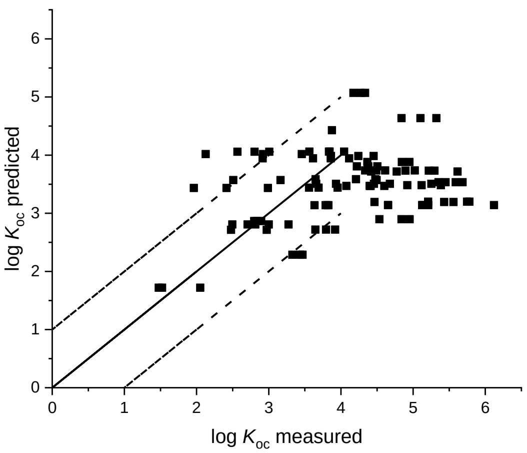

(c)

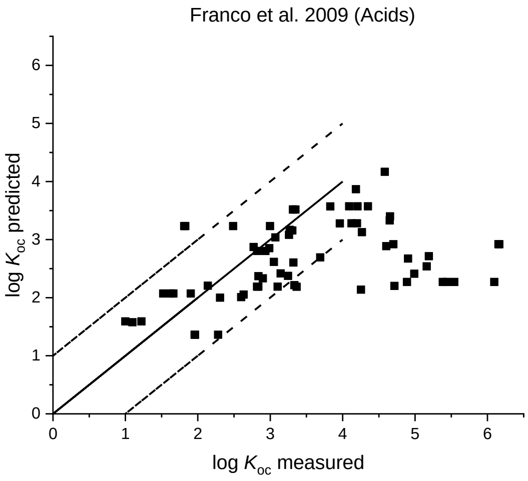

(d)

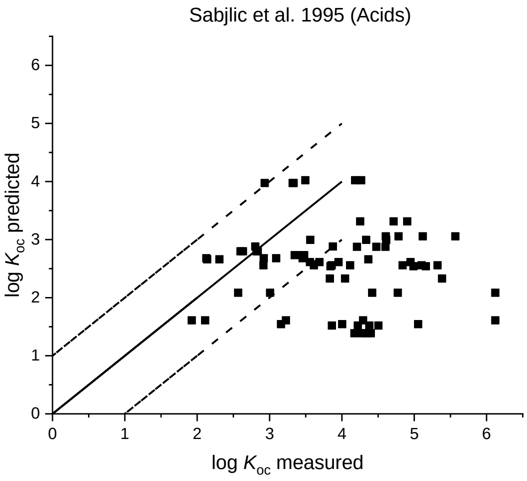

(e)

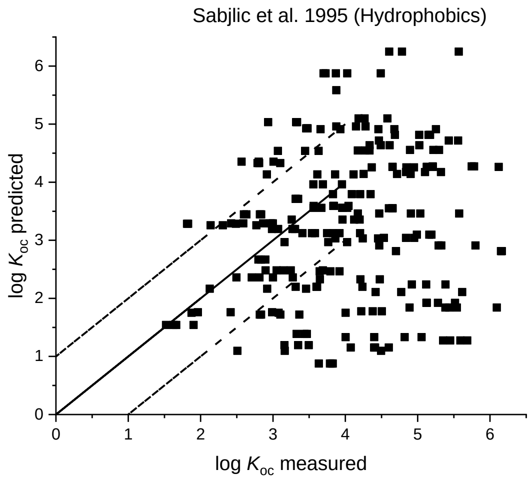

Figure S1. Comparison of measured log K_{oc} values with predicted log K_{oc} values using previously published soil sorption models (Franco et al., [3] acids (**a**); Franco et al., [3] bases (**b**); Franco et al., [4] (**c**); Sabjlic et al., [2] acids (**d**); Sabjlic et al., [2] hydrophobics (**e**)).

(a) **(b)**

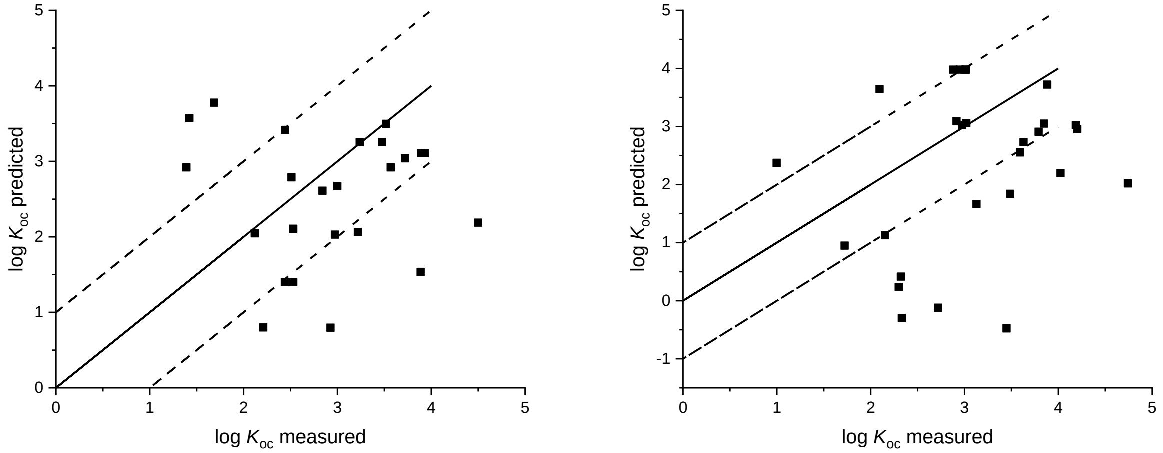

Figure S2. Comparison of measured log K_{oc} values with predicted log K_{oc} values using previously published sludge sorption models (Franco et al., [5] acids (**a**); Franco et al., [5] bases (**b**)).

Table S1. Database of measured soil sorption coefficients provided by EFPIA partners including key physico-chemical properties and soil parameters. All experiments were carried out according to Good Laboratory Practice (GLP) and following OECD 106 [1] or the US FDA Technical Assistance Document 3.08 protocols. Log *Kow*, p*K*a, molecular weight and log Sw (solubility) values were obtained from ACD/I-Labs (v. 2018). Not reported = NR, not applicable = N/A.

Index	Ionisable Functional Group	Molecular Weight (g/mol)	log *Kow*	Acid p*Ka*	Base p*Ka*	Log Sw	% Ionised	log *Kd*	log *Koc*	Soil Type	pH	Clay (%)	Silt (%)	Sand (%)	OC %	CEC (meq/100g)
1	Base	286.33	0.72		5.07	−3.26	0.37	0.62	2.48	Clay loam	7.5	32	31	37	1.4	22.3
2	Base	286.33	0.72		5.07	−3.26	78.79	0.88	2.97	Sandy silt loam	4.5	11	37	52	0.8	8.6
3	Base	349.51	5.7		5.7	−5.54	75.97	3.95	5.43	Clay loam	5.2	29	31	40	3.3	19.2
4	Base	349.51	5.7		5.7	−5.54	16.63	3.31	5.56	Sandy loam	6.4	11	9	80	0.56	9.8
5	Base	349.51	5.7		5.7	−5.54	7.36	2.76	4.47	Sandy loam	6.8	19	15	66	2	15.8
6	Zwitterionic	225.20	−1.76	9.4	2.2	−2.14	0.00	0.54	2.64	Sandy loam	5.2	15.5	17.2	67.3	0.81	14
7	Zwitterionic	225.20	−1.76	9.4	2.2	−2.14	0.00	0.81	2.60	Loam	5.7	55.2	33.2	45.3	1.63	20.1
8	Zwitterionic	225.20	−1.76	9.4	2.2	−2.14	0.00	0.77	2.64	Silt Loam	6.6	21.5	21.5	15.5	1.34	18.5
9	Base	482.62	5.49		12.18; 6.97	−4.77	100.00	2.95	4.33	Silt loam	5.5	11	60	29	4.12	13.2
10	Base	482.62	5.49		12.18; 6.97	−4.28	100.00	2.88	4.28	Loam	5	17	48	35	4	11.4
11	Base	482.62	5.49		12.18; 6.97	−6.12	100.00	2.67	4.17	Sandy Loam	7.4	13	14	73	3.13	17.2
12	Zwitterionic	534.15	5.22	7.62	5.11	−1.44	0.00	1.49	2.81	Clay loam	7.3	NR	NR	NR	4.8	NR
13	Zwitterionic	534.15	5.22	7.62	5.11	−1.44	0.00	1.46	3.10	loam	7.1	NR	NR	NR	2.3	NR
14	Zwitterionic	534.15	5.22	7.62	5.11	−1.44	0.00	0.78	2.79	Sandy loam	6.4	NR	NR	NR	0.98	NR
15	Base	447.15	5.6		7.6	−4.92	99.60	2.18	4.34	Sandy loam	5.2	14	14	72	0.7	6.9
16	Base	447.15	5.6		7.6	−4.92	44.27	3.23	5.02	Sandy clay loam	7.7	27	21	52	1.6	19.6
17	Base	447.15	5.6		7.6	−4.92	28.47	3.12	4.49	Clay loam	8	33	31	36	4.3	17.4
18	Base	447.15	5.6		7.6	−4.92	99.90	2.31	4.61	Loamy sand	4.6	5.7	8.7	85.7	0.5	6.2
19	Base	401.84	4.31		8.64	−3.21	99.94	1.90	3.56	Loamy sand	5.4	6.4	12.2	81.4	2.16	10
20	Base	401.84	4.31		8.64	−3.21	99.43	1.84	3.84	Sandy loam	6.4	9.4	29.8	60.8	0.98	8
21	Base	401.84	4.31		8.64	−3.21	96.50	2.31	3.83	Loam	7.2	27.2	40.6	32.2	2.99	34
22	Base	401.84	4.31		8.64	-3.21	96.50	2.29	4.04	Clay	7.2	42.1	36	21.9	1.75	22
23	Acid	748.29	4.02	7.6; 8.98		−6.61	0.99	2.73	4.20	NR	5.6	31	30	39	3.41	21.5
24	Acid	748.29	4.02	7.6; 8.98		−6.61	2.45	1.89	3.96	NR	6	9	16	75	0.84	9.3
25	Acid	748.29	4.02	7.6; 8.98		−6.61	71.53	1.84	3.26	NR	8	25	24	51	3.76	29.3
26	Acid	748.29	4.02	7.6; 8.98		−6.61	0.13	1.87	4.12	NR	4.7	6	6	88	0.56	4.4
27	Zwitterionic	802.93	4.15	8.78; 9.41	3.9	−4.68	0.00	3.09	4.47	NR	5.5	26	34	40	4.2	22.2

28	Zwitterionic	802.93	4.15	8.78; 9.41	3.9	−4.68	0.00	2.95	4.91	NR	7.9	42	21	37	1.1	31.7
29	Zwitterionic	802.93	4.15	8.78; 9.41	3.9	−4.68	0.00	3.32	5.04	NR	6.4	18	18	64	1.9	19.6
30	Zwitterionic	802.93	4.15	8.78; 9.41	3.9	−4.68	0.00	2.73	4.17	NR	7.9	26	26	48	3.6	30.1
31	Zwitterionic	802.93	4.15	8.78; 9.41	3.9	−4.68	0.00	3.62	5.57	NR	5.4	9	11	80	1.1	11.6
32	Acid	1154.45	4.13	4.34		−6.22	99.83	1.28	2.63	Clay loam	7.1	39.9	36.6	23.5	4.4	25
33	Acid	1154.45	4.13	4.34		−6.22	98.29	1.09	2.84	Sandy clay loam	6.1	24.6	13.5	61.9	1.8	15.7
34	Acid	1154.45	4.13	4.34		−6.22	91.99	0.97	2.82	Sandy loam	5.4	15.6	6.9	77.5	1.4	12.3
35	Acid	1154.45	4.13	4.34		−6.22	99.96	0.50	2.60	Clay loam	7.7	36.5	28.1	35.4	0.8	24.1
36	Acid	366.84	6.17	4.65		−5.55	78.01	2.49	4.58	Sandy loam	5.2	15.5	17.2	67.3	0.81	14
37	Acid	366.84	6.17	4.65		−5.55	91.82	2.40	4.18	Loam	5.7	55.2	33.2	45.3	1.63	20.1
38	Acid	366.84	6.17	4.65		−5.55	98.89	2.39	4.27	Silt Loam	6.6	21.5	21.5	15.5	1.34	18.5
39	Base	696.30	2.64	4.36	6.83	−3.06	98.54	3.88	5.38	NR	5	31	30	39	3.14	19
40	Base	696.30	2.64	4.36	6.83	−3.06	93.10	3.18	5.12	NR	5.7	13	18	69	1.16	11.4
41	Base	696.30	2.64	4.36	6.83	−3.06	34.94	3.16	4.92	NR	7.1	21	12	67	1.74	16.8
42	Acid	408.92	2.04	13.04		−4.00	0.00	1.08	3.08	Sandy loam	6.1	15	21	64	1	10.9
43	Acid	408.92	2.04	13.04		−4.00	0.00	2.24	4.01	Clay loam	5.7	23	47	30	1.7	15.8
44	Acid	408.92	2.04	13.04		−4.00	0.00	−0.22	1.88	Sandy silt loam	4.5	11	37	52	0.8	8.6
45	Base	555.51	7.59		9.04	−4.23	99.98	3.12	4.78	Loamy sand	5.4	6.4	12.2	81.4	2.16	10
46	Base	555.51	7.59		9.04	−5.82	99.10	2.88	4.61	Clay	7	42	36	21.9	1.89	20
47	Base	555.51	7.59		9.04	−6.87	89.70	3.47	5.57	Sandy clay loam	8.1	25	10	65	0.8	16
48	Base	543.46	3.17		2.74	−5.78	0.00	1.07	2.90	Sandy loam	7.2	15	NR	NR	1.5	NR
49	Base	543.46	3.17		2.74	−5.78	0.69	1.42	2.84	Loam	4.9	17	NR	NR	3.8	NR
50	Base	543.46	3.17		2.74	−5.78	0.14	1.58	2.80	Silt loam	5.6	17	NR	NR	6	NR
51	Acid	519.34	1.78	11.01; 11.9; 12.6		−1.35	0.00	−0.09	1.66	Sandy clay loam	6.3	25	11	64	1.76	18.1
52	Acid	519.34	1.78	11.01; 11.9; 12.6		−1.35	0.00	0.21	1.62	Clay loam	5.2	33	23	44	3.82	19.3

53	Acid	519.34	1.78	11.01; 11.9; 12.6		−1.35	0.00	−0.15	1.90	Sandy loam	5.2	15	3	82	0.88	10.9
54	Acid	519.34	1.78	11.01; 11.9; 12.6		−1.35	0.03	0.01	1.52	Sandy clay loam	7.5	31	11	58	3.06	26.7
55	Base	467.30	4.98		8.26	−1.44	99.99	0.61	2.92	Loamy sand	4.3	NR	NR	NR	0.5	6.2
56	Base	467.30	4.98		8.26	−1.44	99.99	1.76	3.86	Sandy loam	4.3	NR	NR	NR	0.8	6.6
57	Base	467.30	4.98		8.26	−1.44	97.86	2.37	4.11	Clay loam	6.6	NR	NR	NR	1.8	22.3
58	Base	467.30	4.98		8.26	−1.44	96.65	2.27	3.61	Sandy clay loam	6.8	NR	NR	NR	4.5	22.6
59	Zwitterionic	571.49	−1.62	0.89; 10.2	3.27	−0.07	99.99	0.39	1.96	Clay	5	41	29	30	2.7	52.5
60	Zwitterionic	571.49	−1.62	0.89; 10.2	3.27	−0.07	100.00	0.22	1.96	Clay loam	5.4	30	39	31	1.8	10.1
61	Zwitterionic	571.49	−1.62	0.89; 10.2	3.27	−0.07	100.00	0.70	2.28	Loam	5.2	26	38	36	2.6	17.2
62	Zwitterionic	534.70	−2.69	2.6; 6.98; 11.74	9.74	−4.10	N/A	0.60	2.56	Sandy loam	5.78	9.1	16.9	74	1.09	6.1
63	Zwitterionic	534.70	−2.69	2.6; 6.98; 11.74	9.74	−4.60	N/A	1.23	3.07	Loam	7.05	14.5	36.6	48.9	1.46	10.3
64	Zwitterionic	534.70	−2.69	2.6; 6.98; 11.74	9.74	−4.60	N/A	1.73	3.45	Clay	7	42	36	21.9	1.89	20
65	Acid	510.47	0.47	2.86; 10.64		−2.36	99.98	−0.40	1.22	Clay loam	6.5	27	42	31	2.4	21.1
66	Acid	510.47	0.47	2.86; 10.64		−2.36	99.94	−1.00	1.00	Sandy loam	6.1	15	21	64	1	10.9
67	Acid	510.47	0.47	2.86; 10.64		−2.36	97.76	−1.00	1.10	Sandy silt loam	4.5	11	37	52	0.8	8.6
68	Base	558.14	5.03		10.63; 7.36	−6.05	99.97	3.54	5.32	Clay	7.1	40.6	37	22.4	1.66	23
69	Base	558.14	5.03		10.63; 7.36	−6.05	100.00	3.07	5.10	Sand	6.23	3.48	5.04	91.48	0.92	8.08
70	Base	558.14	5.03		10.63; 7.36	−6.05	100.00	3.13	4.84	Sandy loam	5.5	6.6	12.1	81.3	1.93	10
71	Base	776.02	4.77		6.69	−4.91	100.00	2.15	3.56	Clay Loam	NR	NR	NR	NR	3.9	NR
72	Base	776.02	4.77		6.69	−4.91	100.00	1.87	3.69	Sandy Loam	NR	NR	NR	NR	1.5	NR
73	Base	776.02	4.77		6.69	−4.91	100.00	2.07	3.95	Sandy Loam	NR	NR	NR	NR	1.3	NR

74	Base	531.31	5.96		9.53	−3.20	99.26	1.89	3.47	Sandy loam	7.4	7	26	67	2.58	13.7
75	Base	531.31	5.96		9.53	−0.90	100.00	2.04	3.46	Loam	5.1	15	40	45	3.82	9.6
76	Base	531.31	5.96		9.53	−1.80	99.97	2.00	3.48	Silt loam	6	7	56	37	3.33	10.9
77	Zwitterionic	615.67	3.54	7.52	3.87	−5.99	0.00	2.20	3.76	Sandy silt loam	6.77	15	61	24	2.7	22.32
78	Zwitterionic	615.67	3.54	7.52	3.87	−5.99	0.00	1.93	4.03	Sand	4.78	4	7	89	0.8	7.36
79	Zwitterionic	615.67	3.54	7.52	3.87	−5.99	0.00	1.74	3.16	Sandy silt loam	6.95	16	45	39	3.8	19.52
80	Zwitterionic	811.81	3.47	8.9; 9.51	5.56; 4.86	−2.46	0.00	3.82	5.29	NR	5.6	31	30	39	3.41	21.5
81	Zwitterionic	811.81	3.47	8.9; 9.51	5.56; 4.86	−2.46	0.00	3.72	5.80	NR	6	9	16	75	0.84	9.3
82	Zwitterionic	811.81	3.47	8.9; 9.51	5.56; 4.86	−2.46	0.00	3.04	4.47	NR	8	25	24	51	3.76	29.3
83	Zwitterionic	811.81	3.47	8.9; 9.51	5.56; 4.86	−2.46	0.00	3.07	5.33	NR	4.7	6	6	88	0.56	4.4
84	Base	389.60	6		0.52		0.00	2.51	4.28	Sandy loam	7.4	15	NR	NR	1.71	NR
85	Base	389.60	6		0.52		0.00	3.38	4.15	Loam	6.3	17	NR	NR	17	NR
86	Base	389.60	6		0.52		0.00	2.99	3.88	Loam	5.4	13	NR	NR	13	NR
87	Base	305.41	5.13		8.06	−3.63	79.55	3.00	4.84	Silt Loam	7.47	20.07	68.23	11.7	1.43	15.69
88	Base	305.41	5.13		8.06	−3.04	93.53	3.23	4.95	Sandy clay	6.9	42	36.1	22	1.9	18
89	Base	305.41	5.13		8.06	−1.74	99.65	2.74	4.37	Loamy sand	5.6	7.9	13.5	79.1	2.36	11
90	Zwitterionic	547.66	3.94	9.94; 12.6	1.82	−4.77	0.00	1.26	2.97	Sandy Loam	6.4	18	18	64	1.9	19.6
91	Zwitterionic	547.66	3.94	9.94; 12.6	1.82	−4.77	0.00	0.98	2.42	Loam	5.5	26	34	40	4.2	22.2
92	Zwitterionic	547.66	3.94	9.94; 12.6	1.82	−4.77	0.00	0.91	2.86	Loamy Sand	5.4	9	11	80	1.1	11.6
93	Zwitterionic	547.66	3.94	9.94; 12.6	1.82	−4.77	0.00	1.62	3.00	Sandy Clay Loam	7.9	26	26	48	3.6	30.1
94	Zwitterionic	547.66	3.94	9.94; 12.6	1.82	−4.77	0.00	0.61	2.59	Clay	7.9	42	21	37	1.1	31.7
95	Base	310.82	6.77		9.87; 2.68	−4.58	100.00	3.27	3.88	Clay loam	5.2	33	23	44	3.82	19.3
96	Base	310.82	6.77		9.87; 2.68	−4.58	99.97	3.02	3.88	Sandy clay loam	6.3	25	11	64	1.76	18.1
97	Base	310.82	6.77		9.87; 2.68	−4.58	99.58	2.65	3.88	Sandy clay loam	7.5	31	11	58	3.06	26.7
98	Base	310.82	6.77		9.87; 2.68	−4.58	100.00	2.99	3.88	Sandy loam	5.2	15	3	82	0.88	10.9
99	Zwitterionic	420.50	−3.65	2.86; 8.51	11.32	−2.05	N/A	-0.25	1.40	Sandy loam	5.7	7.9	14.6	77.5	2.29	11
100	Zwitterionic	420.50	−3.65	2.86; 8.51	11.32	−2.05	N/A	0.26	2.16	Loam	6.8	15.3	36.3	48.4	1.24	11.4

101	Zwitterionic	420.50	−3.65	2.86; 8.51	11.32	−2.05	N/A	0.86	2.58	Clay	6.7	41.5	36.4	22.1	1.9	17
102	Zwitterionic	420.50	−3.65	2.86; 8.51	11.32	−2.05	N/A	0.34	2.18	Silt loam	7.2	16.1	69.4	14.5	1.46	19
103	Acid	315.03	4.46			−1.57	0.00	1.59	3.31	Sandy loam	6.6	18	15	67	1.9	22.1
104	Acid	315.03	4.46			−1.57	0.00	1.60	3.35	Loam	6.6	16	44	40	1.8	14.1
105	Acid	315.03	4.46			−1.57	0.00	1.75	3.32	Clay loam	6.5	32	41	27	2.7	27
106	Acid	564.63	3.7	5.85; 11.06		−4.57	87.62	3.19	5.19	Sandy loam	6.7	8.9	28.7	62.5	0.99	10
107	Acid	564.63	3.7	5.85; 11.06		−4.57	96.00	3.12	4.99	Silt Loam	7.23	24.43	52.1	23.47	1.34	19.61
108	Acid	564.63	3.7	5.85; 11.06		−4.57	93.53	3.24	5.16	Loam	7.01	17.82	44.35	37.83	1.19	12.33
109	Base	358.05	2.79		4.96	−2.04	0.46	1.18	2.49	Clay loam	7.3	33	36	31	4.8	16
110	Base	358.05	2.79		4.96	−2.04	0.72	1.18	2.81	Loam	7.1	25	39	36	2.3	22
111	Base	358.05	2.79		4.96	−2.04	42.01	1.52	3.00	Loam	5.1	21	47	32	3.3	13
112	Base	358.05	2.79		4.96	−2.04	3.50	0.70	2.71	Sandy loam	6.4	9.4	30	61	0.98	8
113	Base	358.05	2.79		4.96	−2.04	0.57	1.51	3.27	Clay	7.2	42	36	22	1.7	22
114	Acid	958.22	3.35	10.4		−6.48	0.01	2.64	4.70	Sand	6.1	4.8	4.69	90.51	0.86	7.6
115	Acid	958.22	3.35	10.4		−6.48	0.01	4.35	6.15	Clay loam	6.3	40	34.77	25.23	1.56	25.05
116	Acid	958.22	3.35	10.4		−6.48	0.02	4.56	6.16	Silt loam	6.6	21.61	61.25	17.14	2.51	19.67
117	Acid	409.15	4.56	10		−1.68	0.13	2.73	4.09	Clay loam	7.1	39.9	36.6	23.5	4.4	25
118	Acid	409.15	4.56	10		−1.68	0.01	2.60	4.35	Sandy clay loam	6.1	24.6	13.5	61.9	1.8	15.7
119	Acid	409.15	4.56	10		−1.68	0.00	2.35	4.21	Sandy loam	5.4	15.6	6.9	77.5	1.4	12.3
120	Acid	409.15	4.56	10		−1.68	0.50	1.73	3.83	Clay loam	7.7	36.5	28.1	35.4	0.8	24.1
121	Base	343.93	5.25		8.63	−4.59	94.44	0.85	2.80	Silt loam	7.4	NR	NR	NR	1.1	10.5
122	Base	343.93	5.25		8.63	−4.59	98.17	0.85	2.57	Sandy clay	6.9	NR	NR	NR	1.9	18
123	Base	343.93	5.25		8.63	−4.59	99.91	1.38	3.01	Loamy sand	5.6	NR	NR	NR	2.36	11
124	Acid	500.57	3.73	13.72		−5.33	0.00	2.41	3.75	Clay loam	7.7	34	29	37	4.6	47.4
125	Acid	500.57	3.73	13.72		−5.33	0.00	2.35	3.92	Silty clay loam	6.3	19	64	17	2.7	26.7
126	Acid	500.57	3.73	13.72		−5.33	0.00	2.16	3.58	Clay loam	5.8	20	47	33	3.8	25.1
127	Acid	500.57	3.73	13.72		−5.33	0.00	2.11	4.20	Loamy sand	4.2	11	5	84	0.8	13.4
128	Acid	500.57	3.73	13.72		−5.33	0.00	1.77	3.54	Kansas	5.8	26	53.3	20.7	1.71	22.1
129	Acid	500.57	3.73	13.72		−5.33	0.00	1.76	3.41	Ohio	5.5	31.3	34	30	2.24	21.7
130	Acid	500.57	3.73	13.72		−5.33	0.00	2.01	3.83	California	7.9	12	31.3	56.7	1.53	38.4
131	Acid	585.64	6.09	3.19		−4.19	99.51	1.51	2.93	Clay loam	5.5	36	34	30	3.8	21.7

132	Acid	585.64	6.09	3.19		−4.19	100.00	1.75	3.33	Sandy loam	7.9	12	31.3	56.7	2.6	38.4
133	Acid	585.64	6.09	3.19		−4.19	99.76	1.78	3.32	Silt loam	5.8	26	53.3	20.7	2.9	22.1
134	Zwitterionic	140.06	−0.61	5	8.5	−0.19	N/A	0.45	2.25	Loamy sand	4.5	6	13	78	1.6	7.6
135	Zwitterionic	140.06	−0.61	5	8.5	−0.19	N/A	0.01	1.81	Sandy clay loam	7	18	30	49	1.6	20.4
136	Zwitterionic	140.06	−0.61	5	8.5	−0.19	N/A	0.08	1.72	Sandy loam	6.7	14	23	50	2.3	20.5
137	Zwitterionic	140.06	−0.61	5	8.5	−0.19	N/A	−0.09	1.76	Sand	6.3	4.3	2.7	91	1.4	8.8
138	Zwitterionic	140.06	−0.61	5	8.5	−0.19	N/A	0.12	2.08	Loam/sandy loam	6.5	8.1	9.9	81	1.1	10.1
139	Zwitterionic	255.23	−2.07	9.37	2.15	−1.87	N/A	0.09	1.53	Clay loam	7.6	NR	NR	NR	3.6	NR
140	Zwitterionic	255.23	−2.07	9.37	2.15	−1.87	N/A	−0.02	2.13	Loamy sand	4.1	NR	NR	NR	0.7	NR
141	Base	440.45	2.92		4.06	−6.79	0.07	2.21	3.65	Clay loam	7.2	24	28	48	3.7	20.8
142	Base	440.45	2.92		4.06	−6.79	0.46	2.22	3.92	Clay loam	6.4	32	31	37	2	18.1
143	Base	440.45	2.92		4.06	−6.66	26.64	1.87	3.79	Sandy Loam	4.5	13	14	73	1.2	6.9
144	Base	589.71	2.48		7.57, 4.87, 3.18	−3.70	98.67	3.15	4.77	Loamy sand	5.7	7.5	13.5	78.2	2.36	11
145	Base	589.71	2.48		7.57, 4.87, 3.18	−3.70	98.67	2.79	4.42	Sandy loam	5.7	7.5	13.5	78.2	2.36	9
146	Base	589.71	2.48		7.57, 4.87, 3.18	−3.70	78.79	3.89	5.62	Clay loam	7	42	36	21.3	1.89	20
147	Base	455.54	5.48		1.93	−5.57	0.02	1.67	3.44	Clay loam	5.7	NR	NR	NR	1.7	NR
148	Base	455.54	5.48		1.93	−5.57	0.01	1.07	3.07	Sandy loam	6.1	NR	NR	NR	1	NR
149	Base	455.54	5.48		1.93	−5.57	0.27	1.53	3.63	Sandy silt loam	4.5	NR	NR	NR	0.8	NR
150	Base	229.26	−0.71		4.25	−1.00	0.56	−0.10	1.52	Clay loam	6.5	27	42	31	2.4	21.1
151	Base	229.26	−0.71		4.25	−1.00	1.39	−0.52	1.48	Sandy loam	6.1	15	21	64	1	10.9
152	Base	229.26	−0.71		4.25	−1.00	35.99	−0.05	2.05	Sandy silt loam	4.5	11	37	52	0.8	8.6
153	Base	581.06	5.14		5.63	−7.31	0.84	3.79	5.13	Clay loam	7.7	34	29	37	4.6	NR
154	Base	581.06	5.14		5.63	−6.25	25.31	3.64	5.21	Silt Clay loam	6.1	19	64	17	2.7	NR
155	Base	581.06	5.14		5.63	−5.67	57.43	3.23	4.65	Clay loam	5.5	20	47	33	3.8	NR
156	Base	581.06	5.14		5.63	−4.23	96.42	4.02	6.12	Loamy sand	4.2	11	5	84	0.8	NR
157	Base	943.48	5.14		5.63	−6.86	0.84	3.79	5.13	Clay Loam	7.7	34	29	37	4.6	NR
158	Base	943.48	5.14		5.63	−6.86	25.31	3.64	5.21	Silt Clay Loam	6.1	19	64	17	2.7	NR
159	Base	943.48	5.14		5.63	−6.86	57.43	3.23	4.65	Clay Loam	5.5	20	47	33	3.8	NR
160	Base	943.48	5.14		5.63	−6.86	96.42	4.02	6.12	Loamy sand	4.2	11	5	84	0.8	NR

161	Acid	435.07	3.82	7		1.98	61.31	1.77	3.07	Clay loam	7.2	32.3	47.6	20.1	5	25.9
162	Acid	435.07	3.82	7		1.98	13.68	1.58	3.31	Sandy clay loam	6.2	20.6	16	63.4	1.9	17.8
163	Acid	435.07	3.82	7		1.98	4.77	1.38	3.27	Loamy sand	5.7	11	7.1	81.9	1.3	12.4
164	Acid	435.07	3.82	7		1.98	83.37	0.83	2.99	Clay	7.7	40.4	22.6	37	0.7	30.8
165	Base	382.88	5.94		4.75	−5.12	26.19	3.04	4.46	Clay loam	5.2	33	23	44	3.82	19.3
166	Base	382.88	5.94		4.75	−5.12	0.07	1.88	3.93	Clay	7.9	45	21	34	0.88	26.8
167	Base	382.88	5.94		4.75	−5.12	2.74	2.92	4.68	Sandy clay	6.3	25	11	64	1.76	18.1
168	Base	382.88	5.94		4.75	−5.12	0.18	2.14	3.66	Sandy clay loam	7.5	31	11	58	3.06	26.7
169	Base	382.88	5.94		4.75	−5.12	26.19	3.20	5.25	Sandy loam	5.2	15	3	82	0.88	10.9
170	Acid	293.72	3.9	4.36		−3.98	99.89	0.99	2.31	Clay loam	7.3	36	44	20	4.8	NR
171	Acid	293.72	3.9	4.36		−3.98	84.60	1.29	2.77	Loam	5.1	21	47	32	3.3	NR
172	Acid	293.72	3.9	4.36		−3.98	98.86	0.22	2.14	Silt Loam	6.3	14	84	2	1.2	NR
173	Base	165.63	−2.31		11.9; 2.98	−1.72	100.00	1.20	3.23	Sandy loam	5.5	6	27	67	0.95	3.7
174	Base	165.63	−2.31		11.9; 2.98	−1.72	100.00	0.48	2.11	Loam	7.2	23	39	38	2.33	26.9
175	Base	165.63	−2.31		11.9; 2.98	−1.72	100.00	0.60	1.93	Clay loam	7.3	33	36	31	4.75	16.4
176	Base	165.63	−2.31		11.9; 2.98	−1.72	100.00	2.82	4.29	Loam	4.9	26	24	50	3.4	20.5
177	Base	165.63	−2.31		11.9; 2.98	−1.72	100.00	1.32	3.09	Clay	7.2	42	36	22	1.7	20
178	Base	233.30	2.55		8.8	−0.16	95.23	1.08	2.92	Silt loam	7.5	20.1	68.2	11.7	1.43	16
179	Base	233.30	2.55		8.8	0.14	97.55	1.70	3.46	Clay	7.2	42.1	36	21.9	1.75	22
180	Base	233.30	2.55		8.8	0.24	99.96	0.46	2.13	Loamy sand	5.4	6.4	12.2	81.4	2.16	10
181	Base	265.16	2.75		7.14, 3.94	−2.98	96.50	2.43	4.48	Sandy loam	5.7	4.6	25.8	69.6	0.9	5.3
182	Base	265.16	2.75		7.14, 3.94	−2.98	99.77	2.59	4.21	Sand	4.5	2.5	5.7	91.8	2.4	11.3
183	Base	265.16	2.75		7.14, 3.94	−2.98	25.75	1.80	3.65	Loam	7.6	15.3	49.3	35.4	1.4	13.1
184	Base	1029.26	0.37		no Pka reported	−2.39	0.00	2.72	4.51	Silty clay loam	4.9	54	18	28	1.6	18
185	Base	1029.26	0.37		no Pka reported	−2.39	0.00	2.68	4.34	Sandy loam	5.8	17	70	13	2.2	9.7
186	Base	1029.26	0.37		no Pka reported	−2.39	0.00	2.75	4.30	Sandy loam	8.2	10	79	11	2.8	12.4
187	Acid	521.43	4.27	14.02		−5.21	0.00	2.14	3.56	Clay loam	5.2	33	23	44	3.82	19.3
188	Acid	521.43	4.27	14.02		−5.21	0.00	1.97	4.03	Sandy loam	5.2	15	3	82	0.88	10.9
189	Acid	521.43	4.27	14.02		−5.21	0.00	2.16	3.67	Sandy clay loam	7.5	31	11	58	3.06	26.7
190	Acid	521.43	4.27	14.02		−5.21	0.00	1.90	3.96	Clay	7.9	45	21	34	0.88	26.8
191	Acid	521.43	4.27	14.02		−5.21	0.00	2.21	3.96	Sandy clay loam	6.3	25	11	64	1.76	18.1

192	Zwitterionic	335.46	1.35	10.64	9.54	−2.99	99.97	1.96	3.35	Clay loam	6	25	37	37	4.12	28
193	Zwitterionic	335.46	1.35	10.64	9.54	−2.99	99.95	1.60	3.49	Sandy loam	6.2	79	58	15	1.28	11.5
194	Zwitterionic	335.46	1.35	10.64	9.54	−2.99	95.63	1.61	3.16	Sandy loam	8.2	10	79	11	2.84	12.4
195	Base	583.99	5.15		5.91, 4.79, 1.98	−5.91	61.86	3.57	5.21	NR	5.7	7.9	14.6	77.5	NR	NR
196	Base	583.99	5.15		5.91, 4.79, 1.98	−5.91	11.41	3.91	5.78	NR	6.8	15	36.1	48.9	NR	NR
197	Base	583.99	5.15		5.91, 4.79, 1.98	−5.91	76.39	4.18	5.75	NR	5.4	44.3	53.3	2.4	NR	NR
198	Base	293.40	2.07		7.96	−4.40	99.83	2.69	4.51	Loam	5.2	26	38	36	1.53	17.2
199	Base	293.40	2.07		7.96	−4.40	99.45	2.22	4.22	Sandy loam	5.7	39	79	11	1	7.6
200	Base	293.40	2.07		7.96	−4.40	99.73	2.40	4.38	Clay loam	5.4	30	39	31	1.06	10.1
201	Base	426.48	1.52		8.15	0.00	99.65	2.36	4.00	Loamy sand	5.7	7.9	14.6	77.5	2.29	11
202	Base	426.48	1.52		8.15	−0.30	95.72	3.19	5.05	Loam	6.8	15	36.1	48.9	1.36	4.8
203	Base	426.48	1.52		8.15	−1.07	78.01	3.44	4.82	Clay loam	7.6	36.4	42.4	21.1	4.13	39.5
204	Base	426.48	1.52		8.15	0.00	99.78	2.68	4.40	Silt loam	5.5	16.8	64.1	19.1	1.95	13.9
205	Zwitterionic	439.50	3.62	9.3	8.4	−3.85	99.80	2.24	3.86	Loamy sand	5.7	7.9	13.5	79.1	2.36	NR
206	Zwitterionic	439.50	3.62	9.3	8.4	−3.85	99.37	2.46	4.45	Sandy loam	6.2	9.2	29.8	61	1.02	NR
207	Zwitterionic	439.50	3.62	9.3	8.4	−3.85	96.17	2.52	4.24	Clay	7	42	36	21.9	1.89	NR
208	Zwitterionic	392.90	2.94	6.4	5.3	−4.11	88.82	1.58	2.90	Clay loam	7.3	33	36	31	4.8	16
209	Zwitterionic	392.90	2.94	6.4	5.3	−4.11	83.37	1.51	3.14	Loam	7.1	25	39	36	2.3	22
210	Zwitterionic	392.90	2.94	6.4	5.3	−4.11	4.77	2.21	3.69	Loam	5.1	21	47	32	3.3	13
211	Zwitterionic	392.90	2.94	6.4	5.3	−4.11	50.00	1.04	3.05	Sandy loam	6.4	9.4	30	61	0.98	8
212	Zwitterionic	392.90	2.94	6.4	5.3	−4.11	86.32	1.48	3.25	Clay	7.2	42	36	22	1.7	22
213	Zwitterionic	700.33	2.25		5.49, 2.29	−6.27	0.00	3.76	5.12	Clay loam	7.1	39.9	36.6	23.5	4.4	25
214	Zwitterionic	700.33	2.25		5.49, 2.29	−6.27	0.00	3.54	5.28	Sandy clay loam	6.1	24.6	13.5	61.9	1.8	15.7
215	Zwitterionic	700.33	2.25		5.49, 2.29	−6.27	0.00	3.27	5.13	Sandy loam	5.4	15.6	6.9	77.5	1.4	12.3
216	Zwitterionic	700.33	2.25		5.49, 2.29	−6.27	0.00	3.42	5.52	Clay loam	7.7	36.5	28.1	35.4	0.8	24.1
217	Base	503.56	1.45		8.7	−3.14	83.37	3.66	5.59	Clay	8	45	16	39	1.18	25.4
218	Base	503.56	1.45		8.7	−3.14	99.80	3.61	5.69	Sandy	6	9	16	75	0.84	9.3
219	Base	503.56	1.45		8.7	−3.14	96.93	3.46	5.35	Sandy loam	7.2	21	17	62	1.29	16.5
220	Base	503.56	1.45		8.7	−3.14	99.92	3.98	5.45	Loam	5.6	31	30	39	3.41	21.5
221	Base	314.40	1.23		8.18; 2.15	−1.53	99.93	2.66	4.49	Silty clay loam	5	53	19	28	1.45	17.5
222	Base	314.40	1.23		8.18; 2.15	−1.53	99.58	1.51	3.16	Sandy loam	5.8	17	70	13	2.2	9.7
223	Base	314.40	1.23		8.18; 2.15	−1.53	75.12	1.00	2.51	Sandy loam	7.7	8	78	14	3.1	9.5
224	Base	482.82	5.5		2.14	−5.67	0.04	3.54	5.30	soil 1	5.5	8.2	15.3	76.5	1.74	10.2

225	Base	482.82	5.5		2.14	−5.67	0.00	2.89	4.89	soil 2	6.8	8.7	28.2	63.1	1	10.7
226	Base	482.82	5.5		2.14	−5.67	0.00	3.44	5.22	soil 3	7.1	40.7	34.5	24.8	1.66	26.9
227	Base	376.45	2.05		6.7	−2.30	61.31	0.34	1.96	Clay loam	6.5	27	42	31	2.4	21.1
228	Base	376.45	2.05		6.7	−2.30	79.92	0.41	2.41	Sandy loam	6.1	15	21	64	1	10.9
229	Base	376.45	2.05		6.7	−2.30	99.37	0.89	2.99	Sandy silt loam	4.5	11	37	52	0.8	8.6
230	Zwitterionic	244.21	−2.26	14.23	0.15	−0.18	0.00	−0.89	0.94	Sandy loam	7.2	15	NR	NR	1.5	NR
231	Zwitterionic	244.21	−1.26	14.23	0.15	−0.18	0.00	−0.82	0.60	Loam	4.9	17	NR	NR	3.8	NR
232	Zwitterionic	244.21	−0.26		0.15	−0.18	0.00	−0.44	0.78	Silt Loam	5.6	17	NR	NR	6	NR
233	Acid	989.56	2.15	10.4			0.01	3.64	5.38	Sandy loam	6.3	25	11	64	1.8	18.1
234	Acid	989.56	2.15	10.4			0.00	3.47	4.89	Loam	5.2	33	23	44	3.8	19.3
235	Acid	989.56	2.15	10.4			0.25	3.38	5.44	Loamy sand	7.8	15	3	82	0.88	9.9
236	Acid	989.56	2.15	10.4			0.13	4.03	5.54	Sandy clay loam	7.5	31	11	58	3.1	26.7
237	Acid	989.56	2.15	10.4			0.00	4.04	6.09	Clay	4.9	45	21	34	0.88	29.9
238	Base	366.42	3.63		4.44	−6.39	4.67	3.22	4.95	Loamy sand	5.75	8.11	10.23	81.66	1.85	9.7
239	Base	366.42	3.63		4.44	−6.41	0.17	3.08	4.84	Clay	7.2	42.1	36	21.9	1.75	22
240	Base	366.42	3.63		4.44	−6.41	0.11	2.64	4.53	Sandy loam	7.4	12.9	6.7	80.4	1.28	12.65
241	Base	269.16	0.96		8.7	−2.49	83.37	1.86	3.79	Clay	8	45	16	39	1.18	25.4
242	Base	269.16	0.96		8.7	−2.49	99.80	1.73	3.81	Sandy loam	6	9	16	75	0.84	9.3
243	Base	269.16	0.96		8.7	−2.49	96.93	1.94	3.83	Sandy clay loam	7.2	21	17	62	1.29	16.5
244	Base	269.16	0.96		8.7	−2.49	99.92	2.17	3.63	Clay loam	5.6	31	30	39	3.41	21.5
245	Zwitterionic	239.31	0.01	10.07	8.94	−0.86	99.94	−2.40	−0.63	Clay loam	5.7	23	47	30	1.7	15.8
246	Zwitterionic	239.31	0.01	10.07	8.94	−0.86	99.86	−2.92	−0.92	Sandy loam	6.1	15	21	64	1	10.9
247	Zwitterionic	239.31	0.01	10.07	8.94	−0.86	100.00	−3.70	−1.60	Sandy silt loam	4.5	11	37	52	0.8	8.6
248	Acid	749.94	4.99	3.77		−4.08	98.54	3.02	4.90	Sandy loam	5.6	13	16	71	1.3	7.9
249	Acid	749.94	4.99	3.77		−3.08	99.85	2.97	4.72	Clay loam	6.6	32.97	32.19	34.84	1.8	22.3
250	Acid	749.94	4.99	3.77		−2.68	99.94	2.88	4.25	Clay Loam	7	28	29	43	4.2	23.5
251	Base	523.32	1.3		8.6	−3.75	99.60	2.68	4.40	Sandy clay loam	6.2	20.6	16	63.4	1.9	17.8
252	Base	523.32	1.3		8.6	−3.75	99.87	2.53	4.41	Loamy sand	5.7	11	7.1	81.9	1.3	12.4
253	Base	523.32	1.3		8.6	−3.75	88.82	2.45	4.60	Clay	7.7	40.4	22.6	37	0.7	30.8
254	Base	523.32	1.3		8.6	−3.75	96.17	2.77	4.08	Clay loam	7.2	32.3	47.6	20.1	5	25.9
255	Base	515.16	7.13		0.76	−6.34	0.00	2.15	3.87	Sandy loam	6.4	18	18	64	1.9	19.6
256	Base	515.16	7.13		0.76	−6.34	0.00	2.35	3.73	Loam	5.5	26	34	40	4.2	22.2
257	Base	515.16	7.13		0.76	−6.34	0.00	2.07	4.03	Loamy sand	5.4	9	11	80	1.1	11.6

258	Base	515.16	7.13		0.76	−6.34	0.00	2.26	3.70	Sandy clay loam	7.9	26	26	48	3.6	30.1
259	Base	515.16	7.13		0.76	−6.34	0.00	2.53	4.49	Clay	7.9	42	21	37	1.1	31.7
260	Acid	679.85	3.93	11.86		−7.40	0.00	0.11	1.83	Sandy loam	5.3	16.61	8.93	74.47	1.9	13.3
261	Acid	679.85	3.93	11.86		−7.40	0.00	1.59	3.00	Clay loam	7.2	22.26	26.13	51.61	3.9	13.5
262	Acid	679.85	3.93	11.86		−7.40	0.00	0.07	1.81	Clay loam	6.6	35.08	28.17	36.75	1.8	22.3
263	Acid	679.85	3.93	11.86		−7.40	0.00	0.19	2.49	Sand	4.3	5.46	6.14	88.4	0.5	6.2
264	Zwitterionic	566.52	2.59	11.79	4.23	−3.56	0.00	2.00	3.31	Clay loam	7.3	33	36	31	4.8	16
265	Zwitterionic	566.52	2.59	11.79	4.23	−3.56	0.00	1.96	3.60	Loam	7.1	25	39	36	2.3	22
266	Zwitterionic	566.52	2.59	11.79	4.23	−3.56	0.00	1.60	3.61	Sandy loam	6.4	9	30	61	0.98	8
267	Zwitterionic	566.52	2.59	11.79	4.23	−3.56	0.00	2.47	4.24	Clay	7.2	42	36	22	1.7	22
268	Zwitterionic	489.93	4.26	6.37	2.45	−6.19	91.46	2.84	4.60	Sandy loam	7.4	15	NR	NR	1.71	NR
269	Zwitterionic	489.93	4.26	6.37	2.45	−6.19	45.98	3.22	4.65	Loam	6.3	17	NR	NR	3.72	NR
270	Zwitterionic	489.93	4.26	6.37	2.45	−6.19	9.68	3.26	4.66	Loam	5.4	13	NR	NR	4.06	NR
271	Base	421.31	1.59		2.17	−5.95	0.00	1.73	3.33	Silt Loam	7.25	21.61	NR	NR	2.51	NR
272	Base	421.31	1.59		2.17	−5.95	0.00	1.63	3.47	Clay loam	7.3	40.69	NR	NR	1.44	NR
273	Base	421.31	1.59		2.17	−5.95	0.04	1.36	3.44	Sandy	5.56	4.57	NR	NR	0.84	NR
274	Zwitterionic	492.24	5.82	11.9	4.17	−2.80	0.00	2.76	4.69	Clay	8	45	16	39	1.18	25.4
275	Zwitterionic	492.24	5.82	11.9	4.17	−2.80	0.00	3.10	5.17	Sandy loam	6	9	16	75	0.84	9.3
276	Zwitterionic	492.24	5.82	11.9	4.17	−2.80	0.00	3.13	5.02	Sandy clay loam	7.2	21	17	62	1.29	16.5
277	Zwitterionic	492.24	5.82	11.9	4.17	−2.80	0.00	3.68	5.15	Clay loam	5.6	31	30	39	3.41	21.5
278	Acid	264.15	2	9.3		−0.57	0.63	1.46	2.82	Clay loam	7.1	39.9	36.6	23.5	4.4	25
279	Acid	264.15	2	9.3		−0.57	0.06	1.36	3.10	Sandy clay loam	6.1	24.6	13.5	61.9	1.8	15.7
280	Acid	264.15	2	9.3		−0.57	0.01	0.98	2.84	Sandy loam	5.4	15.6	6.9	77.5	1.4	12.3
281	Acid	264.15	2	9.3		−0.57	2.45	1.27	3.36	Clay cloam	7.7	36.5	28.4	35.4	0.8	24.1
282	Acid	332.31	−4.13	3.46		−0.99	99.71	−0.46	0.93	Clay loam	6	25	38	37	4.1	28
283	Acid	332.31	−4.13	3.46		−0.99	99.82	−0.70	1.19	Sandy loam	6.2	79	68	15	1.3	11.5
284	Acid	332.31	−4.13	3.46		−0.99	100.00	−0.72	0.83	Sandy loam	8.2	10	79	11	2.8	12.4
285	Zwitterionic	290.10	−2.28	1.38; 3.32; 7.34; 10.08	8.45	0.86	N/A	3.47	1.20	Silt Loam	6.3	14	84	2	1.2	14.3
286	Zwitterionic	290.10	−2.28	1.38; 3.32; 7.34; 10.08	8.45	0.86	N/A	1.36	1.24	Sand	4	4.68	6.3	89	1.1	0.35

287	Zwitterionic	290.10	−2.28	1.38;<br>3.32;<br>7.34;<br>10.08	8.45	0.86	N/A	3.54	0.60	Clay loam	7.3	33.3	36.1	30.7	4.75	1.64

Table S2. Database of measured sludge sorption coefficients provided by EFPIA partners including key physico-chemical properties and sludge parameters. All experiments were carried out according to Good Laboratory Practice (GLP) and following OECD 106 [1] or the US FDA Technical Assistance Document 3.08 protocols.

Index	Ionisable Functional Groups	Molecular Weight (g/mol)	log *Kow*	Acid p*Ka*	Base p*Ka*	% Ionised	log *Kd*	log Koc	pH of System	pH of Sludge	OC %	Organic Matter (%)	CEC (meq/100 g)
1	Acid	748.29	4.02	7.6		20.08	2.93	3.48			28.5		
2	Acid	748.29	4.02	7.6		20.08	2.71	3.24			30		
3	Acid	1154.45	4.13	4.34		95.82	2.40	2.84		5.7	35.9		
4	Acid	430.37	4.94	11		0.01	2.15	NC					
5	Acid	519.34	1.78	11.01		0.01	1.17	1.69	4.83–7.05		30.67		
6	Acid	519.34	1.78	11.01		0.01	1.27	1.80	4.83–7.05		29.83		
7	Acid		2.72	3.95		99.82	1.34	NC					
8	Acid	408.13		12.57		0.00	1.71	NC					
9	Acid	296.15	4.06	4.4		99.51	2.11	NC					
10	Acid	564.63	3.7	5.9		63.47	3.37	3.93		6.14	27.6		
11	Acid	564.63	3.7	5.9		74.25	3.33	3.89		6.36	27.88		95.27
12	Acid	958.22	3.35	10.4		0.04	3.01	3.52	7	6.23	31.23	53.84	
13	Acid	958.22	3.35	10.4		0.04	2.67	3.22	7	6.44	28.4	48.96	
14	Acid	409.15	4.56	10		0.00	3.14	3.57	5.25	5.3	37.3		
15	Acid	297.73	−0.58	8.88		0.21	1.00	1.39	6.2		41	70.6	134
16	Acid	297.73	−0.58	8.88		0.02	1.06	1.42	5.2		43	74	131
17	Acid	206.29	3.72	4.3		99.61	0.93	NC					
18	Acid	435.07	3.82	7		3.83	2.52	2.93	5.6		39.4		98.8
19	Acid	293.72	3.9	4.36		99.52	1.71	2.21	6.68		31.5		
20	Acid	293.72	3.9	4.36		99.47	1.60	2.12	6.63		30		
21	Acid	521.43	4.27	13.84		0.00	3.29	3.72		4.78–7.19	36.87		
22	Acid	392.90	2.94	6.66		68.63	2.28	2.97			20.1		
23	Acid	989.56	2.15	10.4		0.04	2.99	3.51			30.2		
24	Acid	2176.26	−1.25	2.82		99.98	2.10	2.53	6.6		37.5		
25	Acid	2176.26	−1.25	2.82		99.93	2.07	2.44	6		42.2		
26	Acid	264.15	2	9.3		0.01	2.08	2.51		5.3	37.3		

27	Acid	424.44	3.22	5.46		94.62	−0.70	NC					
28	Base	551.76	3.3		9.31	99.41	2.21	NC		6.89–7.08			
29	Base	551.76	3.3		9.31	99.47	2.19	NC		6.37–7.04			
30	Base	408.88	4.16		9.36	99.95	2.89	3.02	6.1	5.6–6.0	73.7	73.7	
31	Base	408.88	4.16		9.36	99.96	2.86	2.97		5.6–6.0	76.2	76.2	
32	Base	408.88	4.16		9.36	99.93	2.87	2.91		5.8–6.2	89.4	89.4	
33	Base	450.50			9.14	99.63	3.89	NC					
34	Base	558.14	5.03		10.6	100.00	3.64	4.20	6.14	7.44	27.6		
35	Base	558.14	5.03		10.6	99.99	3.63	4.19	6.36	7.26	27.88		95.27
36	Base	310.82	6.77		9.9	99.87	2.50	3.01			31		
37	Base	310.82	6.77		9.9	99.87	2.40	2.88			32.9		
38	Base	366.50	3.87		-5	0.00	2.56	2.95	5.9	5.8	41		131
39	Base	366.50	3.87		-5	0.00	2.66	3.02	5.45	5.2	43.9		143
40	Base	358.05	2.79		4.96	0.90	1.40	2.09			20.1		
41	Base	384.25	4.83		1.82	0.00	3.45	NC					
42	Base	343.93	5.25		8.63	99.43	3.34	3.88	6.39	8.1	28.55	49.22	
43	Base	343.93	5.25		8.63	99.43	3.35	3.85	6.39	7.4	31.63	54.53	
44	Base	446.91	4.11		7.6	68.38	3.27	NC					
45	Base	589.71	2.48		7.6	97.55	3.41	3.79	6		42.2	72.8	
46	Base	589.71	2.48		7.6	90.91	3.20	3.63	6.6		37.5	64.6	
47	Base	382.88	5.94		4.75	0.56	3.12	3.59			33.98		
48	Base	165.63	−2.31		11.9	100.00	0.30	1.00			20.1		
49	Base	583.99	5.36		5.9	21.99	3.48	4.02	6.45	6.45	28.55		
50	Base	583.99	5.36		5.9	70.10	4.26	4.74	5.53	7.4	33.37		
51	Base	700.33	2.25		5.5		3.21	3.60	5.6		40.54		
52	Base	503.56	1.45		7.94	3.91	2.98	3.49		4.91–9.33	31		
53	Base	503.56	1.45		7.94	3.91	2.65	3.13		4.91–9.33	32.9		
54	Base	259.35	3.1		9.5	99.84	2.62	NC					
55	Base	383.51	1.57		5.46	5.38	2.53	NC					
56	Base	269.16	0.96		8.7	39.78	1.70	2.15	8.19–8.88		35.45		
57	Base	269.16	0.96		8.7	84.00	1.23	1.72	5.58–7.98		32.19		
58	Base	315.42	−0.14		7.42	83.84	1.50	NC					
59	Base	523.32	1.3		8.6	99.90	7.40	1.27	5.6		39.4		
60	Base	515.16	2.59		4.23	2.19	1.90	2.32	5.88		38.1		
61	Base	515.16	2.59		4.23	1.39	1.90	2.30	6.08		40.2		
62	Base	522.57	1.9		2.9		3.20	NC					
63	Base	475.36	4.54		9.7	0.10	3.92	NC					
64	Base	303.39	0.056		7.59	3.13	2.60	3.00	6.1	7	76.2		

65	Base	303.39	0.056		7.59	2.51	2.20	3.00	6	7	73.7	
66	Base	303.39	0.056		7.59	61.86	3.40	4.50	7.8	7	89.4	
67	Zwitterionic	534.44	4.75	7.62	5.11	0.05	1.85	2.27	6.93		38.1	
68	Zwitterionic	534.44	4.75	7.62	5.11	0.27	1.91	2.31	6.16		40.2	
69	Zwitterionic	802.93	4.15	8.8; 9.41	3.9		1.93	2.44			31.31	
70	Zwitterionic	696.30	2.64	4.6	6.8		3.27	3.76			31.9	
71	Zwitterionic	696.30	2.64	4.6	6.8		3.12	3.60			33.5	
72	Zwitterionic	467.30	4.98	9.8	8.3	99.01	2.04	2.39		6.3	44.8	
73	Zwitterionic	440.45	5.01	2.6; 3.73	4.8	99.99	1.23	NC				
74	Zwitterionic	811.81	3.47	8.9; 9.51	4.86; 5.56		3.16	3.71			28.5	
75	Zwitterionic	345.42	2.43	8.74	4.39		1.68	NC				
76	Zwitterionic	434.47	2.13	8.75	2.21		1.40	NC				
77	Zwitterionic	460.44	-1.5	3.3; 6.86	9	99.96	3.85	NC				
78	Zwitterionic	439.50	3.62	9.3	8.4	98.44	4.03	4.46		6.6	37.5	64.6
79	Zwitterionic	439.50	3.62	9.3	8.4	99.60	4.24	4.61		6	42.2	72.8
80	Zwitterionic	515.16	7.13	12.39	0.76		3.92	4.42			31.31	
81	Zwitterionic	492.24	5.82	11.2	4.17		3.64	4.15	5.77		30.6	
82	Zwitterionic	492.24	5.82	11.2	4.17		3.64	4.13	5.79		31.9	
83	Zwitterionic	290.10	−2.28	1.45	8.45		2.36	2.80			36.1	
84	Zwitterionic	290.10	−2.28	1.45	8.45		2.94	3.43			32.4	

p*Ka*, log K_{ow} and molecular weight were predicted using ACD software (v. 2018). NC = not able to be calculated due to missing organic carbon data.

Table S3. Assessment of the performance of previously published soil sorption models in their ability to predict soil sorption coefficients (log K_{oc}).

Reference	Charge Group Relevant to Model	Correlation (Model)	Model Performance					Number of Data within Model Applicability Domain
			R^2	NSE	RMSE	RMSE/MAE	% within a Factor of 10	
TGD (Sabljic et al., 1995) [2]	Hydrophobic chemicals	Log $K_{OC} = 0.10 + (0.81) \log K_{ow}$	0.02	−1.81	0.60	0.46	46.86	239
TGD (Sabljic et al., 1995) [2]	Acids	Log $K_{OC} = 0.32 + (0.60) \log K_{ow}$	0.01	−2.88	0.55	0.37	38.96	77
Franco and Trapp, 2008 [3]	Acids	Log $K_{OC} = \log (f\text{neutral } 10^{0.54 \log Pn + 1.11} + f\text{ion } 10^{0.11 \log Pn + 1.54})$	0.14	0.58	0.66	0.58	75.00	68
Franco and Trapp, 2008 [3]	Bases	Log $K_{OC} = \log (f\text{neutral } 10^{0.37 \log Pn + 1.70} + f\text{ion } 10^{pKa\ 0.65\ f\ 0.14})$	0.12	0.57	0.70	0.64	56.14	114
Franco and Trapp, 2009 [4]	Acids	$K_{OC} = (10^{0.54 \log Pn + 1.11})/(1 + 10^{(pHsoil - 0.6 - pKa)}) + (10^{0.11 \log Pn + 1.54})/(1 + 10^{(pKa - pHsoil + 0.6)})$	0.16	−0.26	0.70	0.67	66.18	68

Table S4. Assessment of the performance of previously published sludge sorption models in their ability to predict sludge sorption coefficients (log K_{oc}).

Reference	Charge Group Relevant to Model	Correlation (Model)	Model Performance					Number of Data within Model Applicability Domain
			r^2	*NSE*	RMSE	RMSE/MAE	% within a Factor of 10	
Franco et al., 2013 [5]	Acids	$Koc = fn\ 10^{0.54 \log Kown + 1.11} + fion 10^{0.11 \log Kown + 1.54}$	5.3E-05	0.99	0.81	0.82	58	19
Franco et al., 2013 [5]	Bases	$Koc\ base = 10^{0.31 \log Dow + 2.78}$	0.0003	−2.04	0.48	0.31	67	28

Table S5. Pharmaceuticals included in the evaluation of selected soil sorption models (numbers correspond to index numbers provided in Table S1).

Reference	Pharmaceuticals within Applicability Domain	Pharmaceuticals Included in Evaluation (Index Number Provided)
Bintein and Devillers (Acids) [6]	38	32 33 34 35 37 38 65 66 67<br>107 108<br>131 132 133 170 172 248 249 250 268<br>23 24<br>25 26 36 106 161 162 163 164 171<br>208 209<br>210 211 212 269 270
Binetein and Devillers (Bases) [6]	85	18 15 20 22 19 21 40 39 55<br>56 57<br>58 73 72 71 88 89 121 122 123<br>144 145<br>156 160 178 179 180 181 182 199 200<br>198 206<br>207 205 218 219 220 221 222 229 242<br>241 243<br>244 252 251 2 1 4 5 3 16<br>17 41<br>87 112 113 109 111 110 143 142 141<br>146 154<br>155 153 158 159 157 166 169 167 168<br>165 183<br>217 223 228 227 240 239 238 253
Sabljic et al. (Hydrophobic chemicals) [2]	194	32 33 34 35 37 38 107 108 131<br>132 133<br>170 172 248 249 250 268 9 10 11<br>15 18<br>19 20 21 22 39 40 45 46 47<br>55 56<br>57 58 68 69 70 71 72 73 88<br>89 95<br>96 97 98 121 122 123 144 145 156<br>160 178<br>179 180 181 182 198 199 200 205 206<br>207 218

		219 220 221 222 229 241 242 243 244<br>251 252<br>254 23 24 25 26 36 42 43 44<br>51 52<br>53 54 103 104 105 106 114 115 116<br>117 118<br>119 120 124 125 126 127 128 129 130<br>161 162<br>163 164 171 187 188 189 190 191 208<br>209 210<br>211 212 233 234 235 236 237 260 261<br>262 263<br>269 270 278 279 280 281 3 4 5<br>16 17<br>41 48 49 50 84 85 86 87 109<br>110 111<br>112 113 141 142 143 146 147 148 149<br>153 154<br>155 157 158 159 165 166 167 168 169<br>183 217<br>223 224 225 226 227 228 238 239 240<br>253 255<br>256 257 258 259 271 272 273
Sabljic et al. (Acids) [2]	77	32 33 34 35 37 38 107 108 131<br>132 133<br>170 172 248 249 250 268 23 24 25<br>26 36<br>42 43 44 51 52 53 54 103 104<br>105 106<br>114 115 116 117 118 119 120 124 125<br>126 127<br>128 129 130 161 162 163 164 171 187<br>188 189<br>190 191 208 209 210 211 212 233 234<br>235 236<br>237 260 261 262 263 269 270 278 279<br>280 281
Kah and Brown	7	32 33 34 35 170 171 172

Franco and Trapp [3](Acids)	68	32 33 35 34 37 38 59 60 61<br>65 66<br>67 107 108 131 132 133 170 172 248<br>249 250<br>268 23 24 25 26 36 51 52 53<br>54 103<br>104 105 106 114 115 116 117 118 119<br>120 161<br>162 163 164 171 208 209 210 211 212<br>233 234<br>235 236 237 260 261 262 263 269 270<br>278 279<br>280 281
Franco and Trapp [3](Bases)	114	9 10 11 15 18 19 20 21 22<br>39 40<br>55 56 57 58 68 69 70 71 72<br>73 88<br>89 95 96 97 98 121 122 123 144<br>145 156<br>160 178 179 180 181 182 198 199 200<br>205 206<br>207 218 219 220 221 222 223 241 242<br>243 244<br>245 246 247 251 252 254 1 2 3<br>4 5<br>16 17 41 48 49 50 87 109 110<br>111 112<br>113 141 142 143 146 150 151 152 153<br>154 155<br>157 158 159 165 166 167 168 169 183<br>195 196<br>197 217 223 224 225 226 227 228 238<br>239 240<br>253 271 272 273
Franco et al., [4](Acids)	68	32 33 35 34 37 38 59 60 61<br>65 66<br>67 107 108 131 132 133 170 172 248<br>249 250

		268 23 24 25 26 36 51 52 53 54 103 104 105 106 114 115 116 117 118 119 120 161 162 163 164 171 208 209 210 211 212 233 234 235 236 237 260 261 262 263 269 270 278 279 280 281
Droge and Goss [7]	66	15 18 19 20 21 22 55 56 57 58 68 69 70 95 96 97 98 121 122 123 144 145 146 173 174 175 176 177 192 193 194 198 199 200 218 219 220 221 222 241 242 243 244 245 246 247 254 251 252 9 10 11 74 75 76 88 89 201 202 204 178 179 180 45 46 47

Table S6. Pharmaceuticals included in the evaluation of selected sludge sorption models (numbers correspond to index numbers provided in Table S2).

Reference	Pharmaceuticals within Applicability Domain	Pharmaceuticals Included in Evaluation (Index Number Provided)
Franco et al., [5](Acids)	15	1 2 3 10 11 14 15 16 18 19 20 22 24 25 26
Franco et al., [5] (Bases)	28	30 31 32 34 35 36 37 38 39 40 42 43 45 46 47 48 49 50 52 53 56 57 59 60 61 64 65 66
Sathyamoorthy and Ramsburg [8](Acids with a negative charge)	10	3 73 7 9 17 19 20 24 25 27
Sathyamoorthy and Ramsburg [8](Acids)	23	3 73 7 9 17 19 20 24 25 27 5

		6 10 11 12 13 14 15 16 18 21 23 26
Berthod et al. [9](All)	66	28 29 30 31 32 67 68 1 2 69 3 70 71 5 6 72 34 35 74 36 37 38 39 10 11 40 12 13 14 42 43 15 16 45 46 18 47 19 20 48 21 49 50 78 79 22 51 52 53 23 56 57 59 24 25 80 60 61 64 65 66 81 82 26 83 84
Berthod et al. [9] (Acids)	21	1 2 3 5 6 10 11 12 13 14 15 16 18 19 20 21 22 23 24 25 26
Berthod et al. [9] (Bases)	32	28 29 30 31 32 72 34 35 36 37 38 39 40 42 43 45 46 47 48 49 50 51 52 53 56 57 59 60 61 64 65 66
Berthod et al. [9] (Multiple ionisable functional groups)	13	69 67 68 70 71 74 80 78 79 81 82 83 84

References

1. Organisation for Economic Co-operation and Development (OECD). *Test No. 106: Adsorption -- Desorption Using a Batch Equilibrium Method*. *OECD Guidel. Test. Chem. Sect. 1. Phys. Prop.* 2000. OECD Publishing. Available from file:///content/book/9789264069602-en http://dx.doi.org/10.1787/9789264069602-en.
2. Sabljić A: Güsten H, Verhaar H, Hermens J. QSAR modelling of soil sorption. Improvements and systematics of log KOCvs. log KOWcorrelations. *Chemosphere* **1995**, *31*, 4489–4514.
3. Franco A, Trapp S. Estimation of the soil–water partition coefficient normalized to organic carbon for ionizable organic chemicals. *Environ. Toxicol. Chem.* **2008**, *27*, 1995–2004.

4. Franco A, Fu W, Trapp S. Influence of soil pH on the sorption of ionizable chemicals: modeling advances. *Environ. Toxicol. Chem.* **2009**, *28*, 458–464.
5. Franco A, Struijs J, Gouin T, Price OR. Evolution of the sewage treatment plant model SimpleTreat: Applicability domain and data requirements. *Integr. Environ. Assess. Manag.* **2013**, *9*, 560–568.
6. Bintein S, Devillers J. QSAR for organic-chemical sorption in soils and sediments. *Chemosphere* **1994**, *28*, 1171–1188.
7. Droge STJ, Goss K-U. Development and Evaluation of a New Sorption Model for Organic Cations in Soil: Contributions from Organic Matter and Clay Minerals. *Environ. Sci. Technol.* **2013**, *47*, 14233–14241.
8. Sathyamoorthy S, Ramsburg CA. Assessment of quantitative structural property relationships for prediction of pharmaceutical sorption during biological wastewater treatment. *Chemosphere* **2013**, *92*, 639–646.
9. Berthod L, Whitley DC, Roberts G, Sharpe A, Greenwood R, Mills GA. Quantitative structure-property relationships for predicting sorption of pharmaceuticals to sewage sludge during waste water treatment processes. *Sci. Total Environ.* **2017**, *579*, 1512–1520.
